# ATM Regulated PTEN Degradation Is XIAP E3 Ubiquitin Ligase Mediated in p85α Deficient Cancer Cells and Influence Platinum Sensitivity

**DOI:** 10.3390/cells8101271

**Published:** 2019-10-18

**Authors:** Reem Ali, Muslim Alabdullah, Islam Miligy, Makhliyo Normatova, Roya Babaei-Jadidi, Abdolrahman S. Nateri, Emad A. Rakha, Srinivasan Madhusudan

**Affiliations:** 1 Translational Oncology, Division of Cancer and Stem Cells, School of Medicine, University of Nottingham, Nottingham NG7 2RD, UK; mszra2@exmail.nottingham.ac.uk; 2 Department of Pathology, Division of Cancer and Stem Cells, School of Medicine, University of Nottingham, Nottingham NG7 2RD, UK; abdullah.alabdullah@nottingham.ac.uk (M.A.); msxima@exmail.nottingham.ac.uk (I.M.); mrzear1@exmail.nottingham.ac.uk (E.A.R.); 3 Cancer Genetics and Stem Cell Group, Cancer Biology Unit, Division of Cancer and Stem Cells, School of Medicine, University of Nottingham, Nottingham NG7 2RD, UK; Makhliyo.Normatova@nottingham.ac.uk (M.N.); mszrb3@exmail.nottingham.ac.uk (R.B.-J.); mszas8@exmail.nottingham.ac.uk (A.S.N.); 4 Department of Oncology, Nottingham University Hospital, Nottingham NG5 1PB, UK

**Keywords:** ATM, PTEN, CK2α, p85α, XIAP, cisplatin, ovarian cancers

## Abstract

Ataxia-telegiectasia mutated (ATM), phosphatase and tensin homolog (PTEN), and p85α are key tumour suppressors. Whether ATM regulates PTEN expression and influence platinum sensitivity is unknown. We generated ATM knockdowns (KD) and CRISPR knock outs (KO) in glioblastoma (LN18, LN229) and ovarian cancer cells (OVCAR3, OVCAR4). Doxycycline inducible PTEN expression was generated in LN18 and LN229 cells. Transient KD of p85α, CK2, and XIAP was accomplished using siRNAs. Stable p85α knock-in was isolated in LN18 cells. Molecular biology assays included proteasome activity assays, PCR, flow cytometry analysis (cell cycle, double strand break accumulation, apoptosis), immunofluorescence, co-immunoprecipitation, clonogenic, invasion, migration, and 3D neurosphere assays. The clinicopathological significance of ATM, PTEN, p85α, and XIAP (X-linked inhibitor of apoptosis protein) was evaluated in 525 human ovarian cancers using immunohistochemistry. ATM regulated PTEN is p85α dependant. ATM also controls CK2α level which in turn phosphorylates and stabilizes PTEN. In addition, p85α physically interacts with CK2α and protects CK2α from ATM regulated degradation. ATM deficiency resulted in accumulation of XIAP/p-XIAP levels which ubiquitinated PTEN and CK2α thereby directing them to degradation. ATM depletion in the context of p85α deficiency impaired cancer cell migration and invasion reduced 3D-neurosphere formation and increased toxicity to cisplatin chemotherapy. Increased sensitivity to platinum was associated with DNA double strand breaks accumulation, cell cycle arrest, and induction of autophagy. In ovarian cancer patients, ATM, PTEN, p85α, and XIAP protein levels predicted better progression free survival after platinum therapy. We unravel a previously unknown function of ATM in the regulation of PTEN throμgh XIAP mediated proteasome degradation.

## 1. Implication

The study reveals a new function of ataxia-telegiectasia mutated (ATM) in the regulation of phosphatase and tensin homolog (PTEN) throμgh XIAP (X-linked inhibitor of apoptosis protein) E3 ubiquitin ligase mediated proteasome degradation in a p85α dependant.

## 2. Background

Platinating chemotherapeutic agents, such as cisplatin and carboplatin, are frequently used in ovarian cancer therapy [1,2]. Althoμgh the response rates to platinum based chemotherapy can be up to 60–80%, significant toxicities including nausea, vomiting, peripheral neuropathy, nephrotoxicity, and ototoxicity remain a significant clinical problem [1,2]. The cytotoxicity of platinum drμgs is directly related to their ability to interact with DNA and form intra-strand and inter-strand crosslink DNA adducts. If the DNA damaging adducts are not repaired, the cells will accumulate such toxic lesions which lead to cell cycle arrest, generate DNA double strand breaks during replication and ultimately cell death [3]. DNA repair deficient tumour cells will be sensitive to platinum therapy. On the other hand, enhanced DNA repair capacity in tumours is a frequent cause of resistance to therapy which negatively impacts on clinical outcomes. Therefore, the development of predictive biomarkers to personalize ovarian cancer therapy remains a high priority.

Phosphatase and tensin homolog (PTEN) is a key tumour suppressor. The lipid phosphatase activity of PTEN de-phosphorylates PIP_3_ (phosphatidylinositol (3,4,5)-triphosphate) to PIP_2_ (phosphatidylinositol 4,5) triphosphate) and negatively regulates PI3K signalling [4]. Emerging evidence also implicates a role for nuclear PTEN in DNA repair and genomic stability [5,6]. Transcriptional (transcription factor activation, epigenetic silencing), post-transcriptional (miRNA regulation) and post-translational (phosphorylation, de-phosphorylation, oxidation, acetylation, ubiquitination, SUMOylation) mechanisms and protein-protein interactions regulate PTEN functions [reviewed in [7,8,9,10]. PTEN is critically involved in the regulation of cellular proliferation, survival, energy metabolism, cellular architecture and motility [11].

Of the eight mammalian PI3K enzymes grouped into three classes, four class I enzymes (PI3Kα, PI3Kβ, PI3Kγ, and PI3Kδ) have important roles in cancer [12]. PI3K heterodimer consists of a p110 catalytic domain (that phosphorylate PIP_2_ to PIP_3_) and a p85α regulatory sub-unit [13]. In the absence of an activating signal, the interaction of p85α with p110 inhibits p110 kinase activity [12,14]. Receptor tyrosine kinase activation or G-protein coupled receptor activation results in the recruitment of PI3K to the plasma membrane where p85α inhibition of p110 is relieved and p110 phosphorylates PIP_2_ to PIP_3_ leading onto PI3K signal activation. PI3K is a critical regulator of cell proliferation, growth, survival, motility, and metabolism [12,14]. A role for PI3K signalling in nucleotide production required for DNA synthesis during DNA repair has been reported recently [15].

Ataxia-telegiectasia mutated (ATM) is a key member of the PIKK (PI3K-like protein kinases) family of serine/threonine kinases. ATM is a master regulator of DNA damage response following double strand breaks (DSBs) [16]. Following DSBs, ATM is rapidly recruited, converted from homodimers to monomers and auto-phosphorylated. Activated ATM, in turn, phosphorylates a plethora of substrates involved not only in DNA repair but also in cell cycle checkpoints, apoptosis, metabolic and signalling pathways [17,18,19].

PTEN inactivation [20,21], mutations in PIK3R1 (which encodes p85α) that leading to constitutive activation of p110α [8,22,23] and ATM inactivation (through germ-line mutations [24], somatic ATM-inactivating mutations, deletions or epigenetic changes [25,26]) all predispose to cancer. Emerging data also suggests a potential cross-talk between ATM, PI3K, and PTEN pathways. PI3K was previously shown to activate ATM [27]. ATM can also phosphorylate PTEN and promote PTEN nuclear translocation following DNA damage [28]. In addition, p85α can also bind to and increase PTEN lipid phosphatase activity [29].

ATM, PTEN, and p85α are key tumour suppressors. Whether ATM regulates PTEN expression and influence platinum sensitivity in ovarian cancer is unknown. In the current study, we unravel a previously unknown function of ATM in the regulation of PTEN throμgh XIAP E3 ubiquitin ligase mediated proteasome degradation in a p85α dependent manner. ATM depletion in the context of p85α deficiency increased toxicity to cisplatin chemotherapy. Increased sensitivity to platinum was associated with DNA double strand breaks accumulation, cell cycle arrest and induction of autophagy. In ovarian cancer patients, ATM, PTEN, p85α, and XIAP protein levels predicted better progression free survival after platinum therapy.

## 3. Materials and Methods

Compounds and antibodies: KU55933 (ATM kinase inhibitor) was purchased from Tocris Biosciences, Bristol, UK. MK2206 (Akt1inhibitor) was obtained from Selleckchem, Houston, TX, USA. MG132 (proteasome inhibitor) and Cycloheximide (inhibitor of protein synthesis) were obtained from Sigma Aldrich, Gillingham, UK. CK2 inhibitor VIII (calbiochem, Sigma, Aldrich Gillingham, UK). ATM antibody (ab32420), CK2α (ab76040), phospho-CK2α (Thr^360^+Ser^362^) (ab119410). XIAP (ab21278), phospho-XIAP (Ser^87^) (ab175935), p85α (ab86714), phospho-GSK3β (ab131097), total GSK3β (ab76025) and LC3A/B (ab128025) were obtained from Abcam, Cambridge, UK. PTEN antibody (9188s), phospho-PTEN (Ser^380^, Thr^382^, Thr^383^) (9549s), Phospho-AKT1 Ser^473^ (4051S), CyclinD1 (2922s) were purchased from Cell signalling, London, UK. Anti p62 antibody (PM045) was obtained from MBL antibodies, Buckingham, UK. Histone H2AX phosphorylated at Ser^139^ (γH2AX, 05-636, Millipore, Watford, UK).

Cancer cell lines: Glioblastoma cells lines (LN18 and LN229) and epithelial ovarian cancer cell lines (OVCAR3 and OVCAR4) were purchased from American Type Culture Collection (ATCC, Manassas, VA USA) and cultured as per ATCC recommendations. ATM deficient Hela SilenciX and control ATM proficient Hela cells were purchased from Tebu-Bio (www.tebu-bio.com). LN18, LN229, and ATM proficient Hela SilenciX were cultured in Dulbecco’s Modified Eagle Medium (DMEM with 4500 mg/L glucose, L-glutamine, sodium pyruvate) (Sigma, Gillingham, UK) supplemented with 10% FBS and 1% penicillin streptomycin. OVCAR3 and OVCAR4 were cultured in RPMI medium supplemented with L-glutamine, 10% FBS, and 1% penicillin streptomycin. Hela SilenciX cells were grown in Dulbecco’s Modified Eagle’s Medium (with l-glutamine 580 mg/L, 4500 mg/L D19 glucose, with 110 mg/L sodium pyruvate) supplemented with 10% FBS, 1% penicillin/streptomycin, and 125 μg/mL hygromycin B.

ATM knock out (KO) using the CRISPR/Cas-9 system: ATM KO was generated in LN18 and LN229 using the CRISPR/Cas-9 system. Briefly, two pairs of manually designed oligonucleotides as small guide-RNA (sgRNA) were cloned into plasmid vector (PX459-Addgene) expressing puromycin resistance gene as a selection marker. Plasmid was amplified in bacterial competent cells for 24 h and extracted using (medi-prep) kit (Qiagen, Manchester, UK). LN18/LN229 cells were seeded at 50–60% confluency in 12 well plates overnight. 2–3 μg of DNA was delivered using positively charged polyethylenimine compound (PEI) in opti-mem medium. LN18 were selected in 15 μg/mL puromycin. LN229 were selected in 10μg/mL puromycin.

Generation of doxycycline inducible PTEN knockdown by lentiviral shRNA transduction: Doxycycline inducible PTEN knockdown in LN18 and LN299 cell lines by lentiviral shRNA transduction was performed as described previously [30]. Reduced PTEN transcript production was confirmed by endpoint PCR and Western blot analysis.

Transient knockdown of ATM, PI3KR1 (p85α), CK2α and XIAP using siRNAs: ATM siRNA was obtained from Sigma Aldrich, UK. XIAP, Ck2α, PIK3R1 siRNA were obtained from Invitrogen, UK. Transfection was achieved using Lipofecamine 3000 reagent (Invitrogen, Loughborough, UK) as per the manufacturer’s protocol. Briefly, cells were seeded at 50–60% confluency in 10 cm culture dishes transfected in opti-mem low serum media. Cell lysates were collected in RIPA buffer (Sigma, Gillingham, UK) and efficiency of transfection were confirmed using western blotting.

Generation of p85 overexpressing stable (knock-in) LN18 cells: LN18 cells were transfected with PLX304 plasmid expressing ORF sequence of PI3KR1. After 48 h stable clones were selected by geneticin (G418) for 2 weeks. The expression of p85α was confirmed by western blot.

Protein stability assay: Cells were seeded overnight, treated with 100 μg/mL cycloheximide (Sigma) for 4 h and then treated with 100 μM of MG132 (Sigma) and lysates were collected at various time points: 0 h, 1 h, 2 h, and 3 h.

Western blotting: Protein samples were prepared by lysing cells in RIPA buffer (Sigma Aldrich) containing protease inhibitor (Sigma) and phosphatase inhibitor cocktail 2 and 3 (Sigma). Protein quantification was performed using the BCA colorimetric kit (Thermofisher, Altrincham, UK). Samples were run on SDS-PAGE gel (4–12%) bis-tris. Membranes were incubated with primary antibodies (4 °C/overnight), washed and later incubated with infrared dye labeled secondary antibodies (Licor Biosciences, Lincoln, NE, USA) [IRDye 800CW Donkey Anti-Rabbit IgG (H+L) and IRDye 680CW Donkey Anti-Mouse IgG (H+L)] in the dilution of 1:10,000 for 60 min. Protein detection and quantification were determined by scanning the membranes on Licor-Odyssey’s Scanner (Licor Biosciences, Lincoln, NE, USA) at the predefined intensity fluorescence. Where appropriate, AKT1 levels were measured after 24 h of serum starvation. 

Real-time PCR: RNA was extracted using RNeasy Mini kit (Qiagen, Manchester, UK) and cDNA conversion was performed using RT2 first strand kit as per manufacturer’s protocol. Samples were run on the ABI-7500 fast block.

Flow Cytometry analysis: Cells were trypsinized and washed with ice cold PBS, then fixed in 70% ethanol for at least 30 min. After removal of the fixative solution by centrifugation cells were stained with phospho Histone (γH2AX) Ser139. Cells were then treated with RNase and stained with 10 μg/mL propidium iodide (Sigma Aldrich) in PBS. For Apoptosis detection, cells were collected and analysed using annexinV detection kit (BD biosciences, Wokingham, UK). Samples were analysed on the FC500 flow cytometer (Beckman Coulter, Brea, CA, USA) and data were analysed using Weasel software, Version 3.2.1.

Immunofluorescence staining: Cells were seeded on the cover slips overnight then fixed with 4% paraformaldehyde for 30 min and permeabilized with 0.1% triton (Thermofisher) for 30 min. After blocking with 3% BSA cells were incubated with anti PTEN antibody (abcam ab32199) for 1 h at room temperature and then labelled with Goat anti rabbit IgG tetramethylrhodamine (Invitrogen A16129) for 1 h. Slides were prepared in duplicates. Imaging was carried out using a Leica confocal microscope. For analysis, 100 cells per slide were counted.

Co-immunoprecipitation and HA ubiquitination assay: For Ubiquitination assay, LN18 WT and ATM KO Cells were transfected with a plasmid carrying ubiquitin tagged HA for 48 h as described previously [31]. For co-immunoprecipitation, LN229 WT and ATM KO cells were extracted. Cells were resuspended in RIPA buffer containing protease inhibitors on ice for 1 h. Lysates were incubated with the indicated antibodies overnight and then conjμgated to protein A/G magnetic beads for 2 h at room temperature. After IP the beads were washed 4 times thoroughly with Phosphate buffer saline containing 0.01% Tween 20 and protease inhibitors. Immunoprecipitated proteins were eluted using 4 × SDS loading buffer and then boiled at 100 °C for 8 min. Denaturated proteins were separated on 4–12% SDS PAGE.

Caspase 3/7 activity assay: Cells were seeded overnight in 96 well plates, and treated with cisplatin for 48 h. The Caspase-Glo^®^ 3/7 Assay luminogenic substrate was added to the wells for 30 min and caspase 3/7 activity was measured using Luminescence plate reader.

Bio-informatics analyses of phosphorylation sites on CK2α and XIAP: To search for an S/TQ cluster domain in human CK2α and XIAP proteins we used Pearl and Python, available at the following URL: http://ustbioinfo.webfactional.com/scd/.

3D-neurospheres: 4 × 10^4^ cells per well were seeded in ultra-low attachment 6-well plates in promo cell serum free cancer stem cells medium. On day 14 cells were fixed with 4% formaldehyde and stained with 0.001 µM calcein AM and 2.5 µM ethidium bromide homodimer. Images were analysed by Leica software.

Cell proliferation and Clonogenic survival assays: 100 cells were seeded in 96-well plates in triplicates overnight and treated the next day with Cisplatin or Methyl methanesulphonate at the indicated concentrations. Cell viability was measured by cell titer cell proliferation assay (MTS) (Promega, Southampton, UK). For Clonogenic survival assays, 250 cells were seeded in 6-well plates overnight and compounds were added at the indicated concentrations. The plates were left in the incubator for 14 days, after incubation colonies were washed with PBS and stained with crystal violet, acetic acid and methanol mixture then counted. 

Invasion and migration assays: Cells were seeded in the Upper chamber of polycarbonate membrane inserts (8 µm pore size), (Cell biolabs, Cambridge, UK) in serum free medium and left to migrate toward 10% serum containing medium for 24 h. After 24 h medium containing non-invasive cells were aspirated from the inserts and the inner was washed with distilled water then stained with crystal violet for 10 min. Cells were extracted, and 100 µL from each sample was transferred to a 96-well microtiter plate for measuring OD at 560 nm. For Migration assays Cells were seeded in 96 well plates containing hydrogel spot non migratory area, left to adhere overnight and then hydrogel area was digested and cells were left to migrate for 16 h. Then the wells were washed three times, fixed and stained with crystal violet. Cell migration images were analysed by image j software.

Statistical analysis: Statistical data are presented as mean ± SD of at least three independent biological experiments. *P* values were calculated with either the Student two-tailed *t* test and one way ANNOVA for normally distributed datasets or the nonparametric Mann–Whitney two-tailed *U* test.

Bio-informatics analyses of phosphorylation sites on CK2α and XIAP: To search for an S/TQ cluster domain in human CK2α and XIAP proteins we used Pearl and Python, available at the following URL: http://ustbioinfo.webfactional.com/scd/.

Clinical study: Investigation of the expression of ATM, PTEN, p85, and XIAP in ovarian epithelial cancer was carried out on tissue microarrays of 525 consecutive ovarian epithelial cancer cases treated at Nottingham University Hospitals (NUH) between 1997 and 2010. Patients were comprehensively staged as per the International Federation of Obstetricians and Gynaecologists (FIGO) Staging System for Ovarian Cancer. Patient demographics are summarized in Supplementary Table S1. All patients received platinum based chemotherapy. Platinum resistance was defined as patients who had progression during first-line platinum chemotherapy or relapse within 6 months after completion of platinum treatment. Survival was calculated from the operation date until the 1st of October 2016 when any remaining survivors were censored. Progression-free survival was calculated from the date of the initial surgery to disease progression or from the date of the initial surgery to the last date known to be progression-free for those censored. Supplementary methods summarize immunohistochemical protocols, evaluation of immune staining and statistical analyses. Tumour Marker Prognostic Studies (REMARK) criteria, recommended by McShane et al. [32], were followed throμghout this study. This work was approved by Nottingham Research Ethics Committee.

Investigation of the expression of p85α, ATM, PTEN, and XIAP in ovarian epithelial cancer was carried out on tissue microarrays of 525 consecutive ovarian epithelial cancer cases treated at Nottingham University Hospitals (NUH) between 1997 and 2010. Patients were comprehensively staged as per the International Federation of Obstetricians and Gynaecologists (FIGO) Staging System for Ovarian Cancer. Survival was calculated from the operation date until the 1st of October 2016 when any remaining survivors were censored. Patient demographics are summarized in Supplementary Table S1. Platinum resistance was defined as patients who had progression during first-line platinum chemotherapy or relapse within 6 months after completion of platinum treatment.

Tissue microarrays (TMAs) were constructed as described previously [1]. Briefly, triplicate tissue cores with a diameter of 0.6mm were taken from the tumour and arrayed into a recipient paraffin block using a tissue puncher/arrayer (Beecher Instruments, Silver Spring, MD, USA) as previously described [1]. Four micron sections of the tissue array block were cut and placed on Surgipath X-tra Adhesive microscope slides (Leica Microsystems, Wetzlar, Germany) for immunohistochemical staining. Immunohistochemical staining for P85, ATM, PTEN, and XIAP was performed using Thermo Scientific Shandon Sequenza chambers and the Leica Novolink max polymer detection system (RE7280-K) according to manufacturer instructions (Leica Microsystems). Pre-treatment of TMA sections was performed with citrate or EDTA buffer (pH 6.0, 20 min, Microwave or PH 9.0 hot water bath respectively). TMA sections were incubated at room temperature with each antibody according to optimal conditions and summarized in Supplementary Table S2. Negative controls with no primary antibody were included in each run.

The tumour cores were evaluated by expert pathologists blinded to the clinico-pathological characteristics of patients. Whole field inspection of the core was scored, the sub cellular localisation of each marker was identified (nuclear, cytoplasm, cell membrane), and the optimal scoring methodology was applied in each case (summarized in Supplementary Table S2). Intensities of subcellular compartments were each assessed and grouped as follows: 0 = no staining, 1 = weak staining, 2 = moderate staining, 3 = strong staining. The percentage of tumour cells in each category was estimated (0–100%). H-score (range 0–300) was calculated by multiplying the intensity of staining and the percentage of staining. Not all cores within the TMA were suitable for IHC analysis due to missing cores or absence of tumour cells.

Statistical analysis was performed using SPSS v 22 (IBM, Chicago, IL, USA) for Windows. Association with clinical and pathological parameters using categorised data was examined using a Chi-squared test. All tests were 2-tailed. The median was utilised to define the single optimal cut-off point for H score. Survival rates were determined using Kaplan–Meier method and compared by the log-rank test. All analyses were conducted using Statistical Package for the Social Sciences (SPSS, version 22) software for windows. A *p* value of less than 0.05 was identified as statistically significant. This work was approved by the Nottingham Research Ethics Committee.

## 4. Results

**Figure 1:**
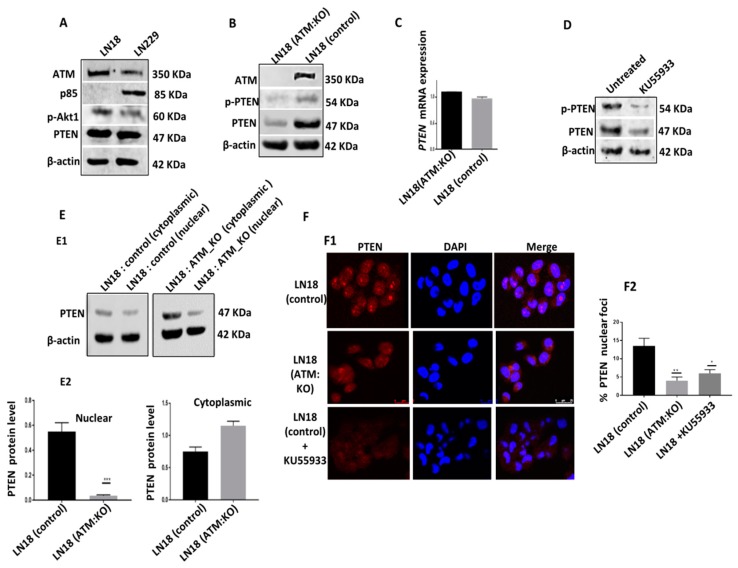
Low PTEN protein level in ATM deficient cells. (**A**) ATM, PTEN, p-AKT1 and p85α levels in LN18 and LN229 cells. (**B**) LN18 ATM_KO was generated by CRISPR-cas9 and levels of total PTEN and P-PTEN (Ser380/Thr382/383) were assessed by western blotting. (**C**) Relative PTEN mRNA expression was assessed by RT-PCR. (**D**) LN18 control cells were treated with ATM inhibitor KU55933 for 24 h lysates were immunoblotted for PTEN and p-PTEN. (**E**) Nuclear and cytoplasmic fractionation of LN18 control and ATM KO cells. (**F**) Representative photomicrograph images showing PTEN translocation (100×). The data are representative of 3 independent experiments. Where appropriate, quantification is summarized in Supplementary Figure S1.

ATM depletion or inhibition and PTEN degradation in p85α deficient cells: As shown in Figure 1A, the LN18 cell line is p85α deficient compared to the LN229 cell line which is p85α proficient. Using CRISPR/Cas-9 system we generated ATM KO cells. In whole cell extracts, in contrast to LN18 control cells, LN18: ATM KO cells have minimal PTEN and p-PTEN levels (Figure 1B, Supplementary Figure S1B). There were no significant changes in *PTEN* mRNA expression as analysed by qRT-PCR in LN18 controls and LN18:ATM KO cells (Figure 1C). Similarly, KU55933 (ATM inhibitor) treatment in LN18 control cells also leads to substantial depletion of PTEN and p-PTEN levels compared to untreated controls. (Figure 1D, Supplementary Figure 1D). We then evaluated PTEN levels in nuclear and cytoplasmic fractions. As shown in Figure 1E, ATM depletion or inhibition results in substantial reduction in nuclear PTEN and its cytoplasmic translocation in LN18:ATM KO cells compared to LN18 control cells. By immunofluorescence microscopy, we also observed significant reduction in PTEN nuclear foci in LN18: ATM KO cells and in KU55933 treated LN18 control cells (Figure 1F).

**Figure 2:**
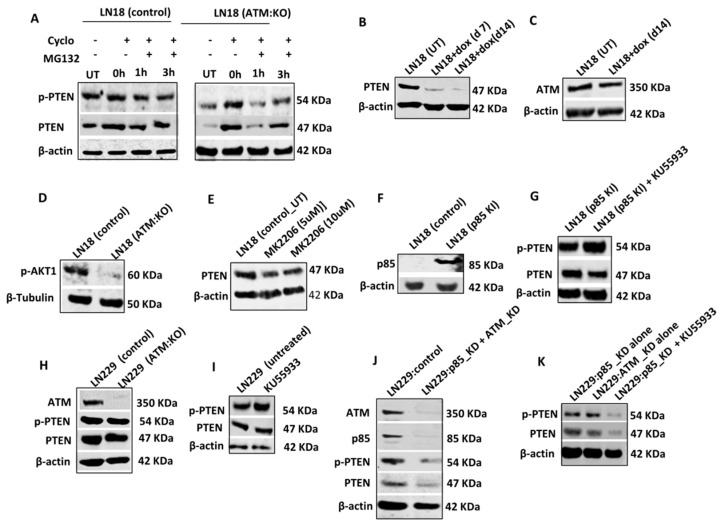
Proteasome mediated PTEN degradation in ATM deficient cells. (**A**) Protein stability assay in LN18 control and ATM KO cells. (**B**) Levels of PTEN and p-PTEN and p85α in Hela ATM SilenciX cells. (**C**) Levels of PTEN and p-PTEN in Hela control cells treated with 10 µM KU55933. (**D**) PTEN and p85α levels in OVCAR3 and OVCAR4 ovarian cell lines. (**E**) PTEN and p-PTEN levels in ATM depleted OVCAR3 cells. (**F**) PTEN and p-PTEN levels in OVCAR3 were treated with 10 µM of KU55933. (**G**) Generation of doxycycline inducible PTEN knock downs. (**H**) ATM level in PTEN Knock downs. (**I**) p-AKT1 levels in LN18 control and ATM KO cells. (**J**) MK2206 pan AKT inhibitor did not affect PTEN levels in LN18 cells. (**K**) p85α overexpression in LN18 control cells (LN18 p85α KI). (**L**) PTEN and p-PTEN levels in LN18 p85α KI cells treated with 10 µM of KU55933. (**M**) PTEN and p-PTEN levels in LN229 ATM KO cells. (**N**) Levels of PTEN, P-PTEN in LN229 cells treated with 10 µM KU55933. (**O**) PTEN and p-PTEN levels in LN229 cells were transfected with p85 SiRNA and ATM SiRNA. (**P**) p85 knock down alone or ATM SiRNA alone did not affect PTEN and p-PTEN levels while p85 knock down and KU55933 leads to PTEN and p-PTEN degradation. (**Q**) PTEN and p-PTEN levels in OVCAR4 cells transfected with ATM SiRNA. (**R**) Levels of PTEN and p-PTEN in OVCAR4 treated with 10 µM of KU55933. (**S**) OVCAR4 cells were transfected with P85 SiRNA alone or ATM SiRNA alone or P85 and ATM SiRNA simultaneously, or p85 SiRNA and 10 µM KU55933. Levels of PTEN and p-PTEN were analysed by western blot. All the data are representative of 3 or more independent experiments. Where appropriate, quantification is summarized in Supplementary Figure S2.

To evaluate whether PTEN that is sequestered in the cytoplasm is then directed to proteasome mediated degradation, we conduced protein degradation assays (Figure 2A, Supplementary Figure S2A). Cells were seeded overnight, treated with 100 μg/mL Cycloheximide (protein synthesis inhibitor) for 4 h and then treated with MG132 (proteasome inhibitor) and sampled at various time points. In untreated cells, at baseline, PTEN levels were low in LN18:ATM KO cells. After 4 h of cycloheximide treatment, PTEN levels in LN18:ATM KO and LN18 control cells were similar. However, within 1 h of MG132 treatment, PTEN levels dropped and then re-accumulated over 3 h in LN18:ATM KO cells. We also observed a similar accumulation of p-PTEN at 3 h in LN18:ATM KO cells. There was no change in PTEN levels in LN18 controls cells (Figure 2A). However, in KU55933 treated LN18 controls cells, within 1 h of MG132 treatment PTEN levels dropped and then re-accumulated over 3 h (Supplementary Figure S7A). We further validated our observations in HeLa and ovarian cancer cell lines. As shown in Supplementary Figures S8A and S4A, p85α deficient ATM KD HeLa cells have reduced levels of PTEN and p-PTEN compared to HeLa control cells. Similarly, KU55933 treated HeLa control cells also demonstrated reduced PTEN and p-PTEN levels (Supplementary Figures S8A and S4A). In OVCAR3 cells which is p85α deficient (Supplementary Figure S8B), ATM depletion or KU55933 treatment leads to reduced PTEN and p-PTEN levels (Supplementary Figures S8C and S4B).

To explore whether PTEN depletion would alter ATM levels, we generated doxycycline inducible PTEN knock down LN18 cells (Figure 2B) but did not observe any alterations in ATM levels in PTEN deficient LN18 cells (Figure 2C). Althoμgh ATM depletion resulted in reduced pAkt1 levels (Figure 2D, Supplementary Figure S2D), MK2206 (Akt1 inhibitor) treatment did not alter PTEN levels (Figure 2E, Supplementary Figure S2E).

p85α protects PTEN from ATM mediated degradation: p85α was previously shown to bind to PTEN and increase PTEN lipid phosphatase activity [29]. In addition, Cheung et al. have also shown that p85α can promote PTEN stability [22]. We, therefore, hypothesised that p85α may be protecting PTEN from ATM regulated degradation in p85α proficient cells. We first generated p85α overexpression stable (knock-in) LN18 cells (Figure 2F). KU55933 treatment in p85α knock-in LN18 cells did not alter PTEN levels in contrast to p85α deficient wild-type LN18 cells (Figure 2G, Supplementary Figure S2G). In addition, in p85α proficient LN229 cells, ATM KO (Figure 2H, Supplementary Figure S2H) or KU55933 (Figure 2I, Supplementary Figure S2I) treatment did not alter PTEN and p-PTEN levels. Doxycycline inducible PTEN knockdown also did not alter ATM levels in LN229 cells (Supplementary Figure S7B). However, in p85α knockdown LN229 cells, ATM depletion (Figure 2J, Supplementary Figure S2J) or KU55933 (Figure 2K, Supplementary Figure S2K) treatment resulted in loss of PTEN and p-PTEN levels. Similarly in p85α proficient OVCAR4 cells, ATM KD or KU55933 treatment (Supplementary Figure S8D) did not alter PTEN and p-PTEN level. However, in p85α knockdown OVACR4 cells, ATM depletion or KU55933 treatment resulted in loss of PTEN and p-PTEN levels (Supplementary Figure S9B).

Taken together, the data provide evidence that ATM is involved in the regulation of proteasome mediated PTEN degradation and p85α may protect PTEN from ATM regulated degradation. To understand molecular pathways involved in this phenomenon, we conducted further mechanistic studies.

**Figure 3:**
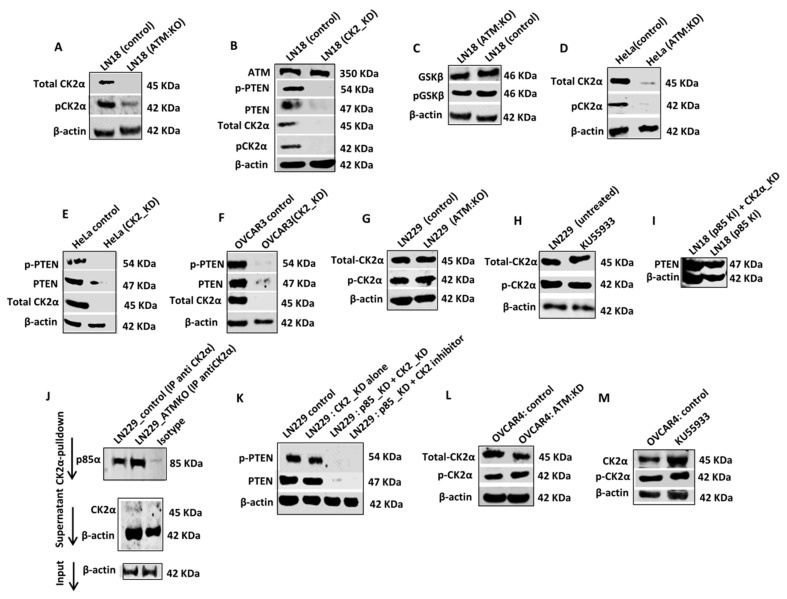
Role of CK2α in ATM mediated PTEN degradation. (**A**) CK2α, p-CK2α levels in LN18 control and LN18 ATM KO cells. (**B**) CK2α, p-CK2α levels in LN18 untreated and KU55933 treated cells. (**C**) CK2α, p-CK2α, PTEN, p-PTEN and ATM levels in LN18 cells were transfected with CK2α SiRNA. (**D**) PTEN, p-PTEN levels in LN18 cells treated with CK2α inhibitor. (**E**) Levels of GSK3β and p-GSK3β in LN18 control and ATM KO cells. (**F**) Levels of CK2α, P-CK2α in Hela control and Hela ATM KO. (**G**) CK2α and p-CK2α levels in Hela control cells treated with KU55933. (**H**) PTEN, p-PTEN and CK2α levels in Hela control cells transfected with CK2α SiRNA. (**I**) Levels of PTEN, p-PTEN in Hela cells treated with CK2α inhibitor. (**J**) PTEN, p-PTEN and CK2α levels in OVCAR3 cells transfected with CK2α SiRNA. (**K**) PTEN, p-PTEN and CK2α levels in OVCAR3 cells treated with CK2α inhibitor. (**L**) CK2α and p-CK2α levels in LN229 ATM KO cells. (**M**) CK2α and p-CK2α levels in LN229 cells treated with KU55933 (10 µM). (**N**) p85 coimmunoprecipitated with CK2α protein. (**O**) PTEN and p-PTEN levels in LN229 cells transfected with p85 SiRNA alone or CK2α SiRNA alone or P85 and CK2α SiRNA simultaneously. (**P**) OVCAR4 cells were transfected with ATM SiRNA. Levels of CK2α, p-CK2α were analysed by western blot on day 3. (**Q**) CK2α and p-CK2α levels in OVCAR4 cells treated with KU55933 (10µM). (**R**) PTEN, p-PTEN and p85α levels in OVCAR4 cells transfected with p85 SiRNA alone or CK2α SiRNA alone or P85α and CK2α SiRNA simultaneously. (**S**) PTEN level in LN18 p85KI transfected with CK2 SiRNA. All figures are representative of 3 independent experiments. Where appropriate, quantification is summarized in Supplementary Figure S3.

ATM regulates Casein Kinase 2α (CK2α) levels in p85α deficient cells: CK2α, a messenger-independent serine threonine kinase can phosphorylate C-terminus of PTEN (at residues 369–386), promote PTEN stability and prevent proteasome mediated PTEN degradation [33]. We speculated that ATM may regulate CK2 levels. In LN18:ATM KO cells (Figure 3A, Supplementary Figure S3A) or in KU55933 treated LN18 control cells (Supplementary Figures S8E and S5A), there were a significant reduction of CK2α and pCK2α levels. Moreover, CK2α KD (Figure 3B, Supplementary Figure S3B) or treatment with CK2 inhibitor in LN18 control cells (Supplementary Figures S8F and S5B) also leads to loss of PTEN and p-PTEN levels. GSK3β was previously shown to be involved in PTEN phosphorylation and stabilization [34]. However, there was no change in GSK3β or pGSK3β levels in LN18 ATM KO or KU55933 treated LN18 cells (Figure 3C, Supplementary Figure S3C). In HeLa cells, ATM KD (Figure 3D, Supplementary Figure S3D) or KU55933 treatment (Supplementary Figures S8G and S5C), lead to loss of CK2α and pCK2α levels. In addition, in HeLa cells, CK2α KD (Figure 3E, Supplementary Figure S3E) or CK2 inhibitor treatment (Supplementary Figures S8G and S5D) also leads to the loss of PTEN and p-PTEN levels. In OVCAR3 cells, similarly, CK2α KD (Figure 3F, Supplementary Figure S3F) or CK2 inhibitor treatment (Supplementary Figures S8H and S5E), resulted in loss of PTEN and p-PTEN levels.

In LN229 cells, ATM KO alone did not affect CK2α and pCK2α levels (Figure 3G, Supplementary Figure S3G). In co-IP experiments, we observed that CK2α physically interacted with p85α (Figure 3J) sμggesting that p85α may also be protecting CK2α from ATM mediated degradation. Accordingly, p85α KD with either CK2α KD or CK2 inhibitor treatment resulted in the loss of PTEN and p-PTEN levels in LN229 cells (Figure 3K, Supplementary Figure S3K). In OVCAR 4 cells, similarly, ATM KO alone (Figure 3L, Supplementary Figure S3L) or KU55933 treatment (Figure 3M, Supplementary Figure S3M) did not affect CK2α and pCK2α levels but p85α KD with either CK2α KD or CK2 inhibitor treatment, resulted in loss of PTEN and p-PTEN levels (Supplementary Figure S9C). Moreover, CK2α depletion in p85α knock-in LN18 cells did not alter PTEN levels (Figure 3I) in contrast to p85α deficient wild-type LN18 cells (Figure 3I, Supplementary Figure S3I).

The data, therefore, provide evidence that CK2α is involved in ATM regulated PTEN degradation in p85α deficient cells. In addition, p85α protected CK2α from ATM mediated degradation in p85α proficient cells. We then proceeded to identify the E3 ubiquitin ligase that may be involved in directing proteasome mediated degradation of PTEN and CK2α.

**Figure 4:**
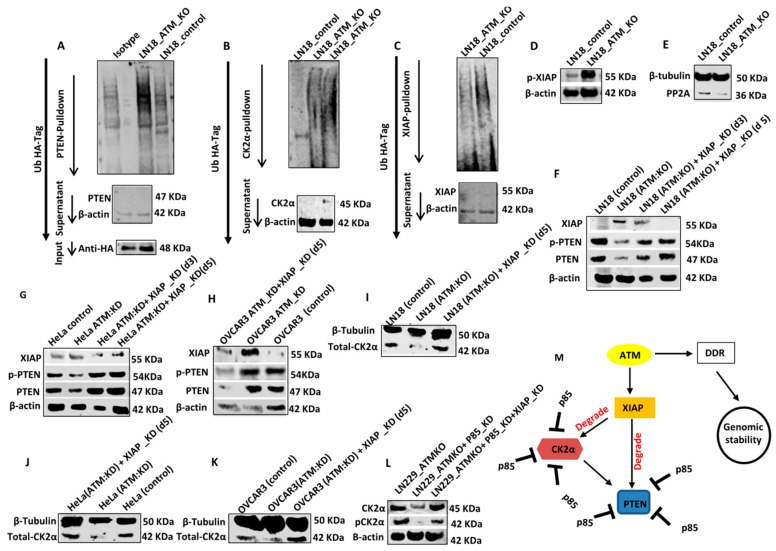
XIAP E3-Ubiquitin ligase and ATM mediated PTEN degradation. (**A**) PTEN IP was blotted on SDS-PAGE gel against HA antibody. (**B**) CK2 IP was blotted on SDS-PAGE against HA antibody. (**C**) XIAP IP was blotted on SDS-PAGE against HA antibody. (**D**) p-XIAP levels in LN18, LN229 control, and ATM KO. (**E**) Levels of PP2A by western blot in LN18 and LN229 control and ATM_KO (**F**). LN18 ATM KO cells were transfected with XIAP SiRNA. Levels of PTEN, p-PTEN and XIAP were analysed by western blot on day3 and day5. (**G**) Hela ATM _KD cells were transfected with XIAP SiRNA. Levels of PTEN, p-PTEN and XIAP were analysed by western blot on day3 and day5. (**H**) Levels of PTEN, p-PTEN and XIAP in OVCAR3 cells transfected with ATM SiRNA alone or ATM SiRNA and XIAP SiRNA Simultaneously. (**I**) LN18 ATM KO was transfected with XIAP SiRNA. On day5 levels of CK2α were analysed. (**J**) Hela ATM _KD cells were transfected with XIAP SiRNA. On day5 levels of CK2α were analysed. (**K**) OVCAR3 cells were transfected with ATM SiRNA and XIAP SiRNA simultaneously. On day5 levels of CK2α were analysed. (**L**) LN229 ATM KO cells were transfected with p85 SiRNA alone or p85 SiRNA and XIAP SiRNA simultaneously. Levels of CK2α and p-CK2α were analysed on day5. All data are representative of 3 independent experiments. Where appropriate, quantification is summarized in Supplementary Figure S4. (**M**) Proposed model for ATM regulated PTEN degradation.

CK2α and PTEN degradation are XIAP (X-linked inhibitor of apoptosis protein) mediated and ATM regulated: Polyubiquitination of PTEN results in proteasome mediated degradation of PTEN protein [7,8,9]. We, therefore, investigated if ATM knockout induces polyubiquitination of PTEN. For ubiquitination assay, LN18 control cells and LN18: ATM KO cells were transfected with a plasmid carrying ubiquitin tagged HA as described previously [31]. Lysates were then incubated with PTEN antibody, conjμgated with A/G magnetic beads, protein eluted and immunoblotted with anti-HA antibody. As shown in Figure 4A, ATM KO resulted in polyubiquitination of PTEN compared to control cells. The polyubiquitination of CK2α was also evident in ATM KO cells compared to control cells (Figure 4B). The E3 ubiquitin ligase activity of XIAP was previously shown to induce polyubiquitination of PTEN [35]. We, therefore, speculated that ATM may regulate XIAP levels. In LN18 control cells, we observed high basal levels of polyubiquitinated XIAP whereas in LN18: ATM KO cells polyubiquitinated XIAP was significantly reduced (Figure 4C). The data sμggested that ATM regulates polyubiquitination of XIAP. In addition, we also observed an accumulation of pXIAP in LN18:ATM KO cells (Figure 4D) which were interestingly associated with reduction in the levels of PP2A protein phosphatase (Figure 4E, Supplementary Figure S6E), a key regulator of cellular protein phosphorylation in cells. As expected, XIAP levels accumulated in LN18:ATM KO cells compared to LN18 control cells (Figure 4F, Supplementary Figure S6F). We also confirmed that XIAP is also induced in HeLa ATM:KO cells (Figure 4G, Supplementary Figure S6G) and OVCAR3 cells (Figure 4H, Supplementary Figure S6H). Induction of XIAP expression was associated with a corresponding reduction in PTEN and pPTEN levels in LN18:ATM KO cells (Figure 4F, Supplementary Figure S6F), HeLa ATM:KD cells (Figure 4G, Supplementary Figure S4G,) and OVCAR3 cells (Figure 4H, Supplementary Figure S6H). Importantly, upon depletion of XIAP by siRNA in LN18:ATM KO, PTEN, and pPTEN levels were restored to the levels observed in control cells (Figure 4F, Supplementary Figure S6F). A similar observation was confirmed in HeLa ATM:KD cells (Figure 4G, Supplementary Figure S6G) and OVCAR3 cells (Figure 4H, Supplementary Figure S6H).

We then investigated whether XIAP also mediated CK2α degradation. Compared to LN18 control cells, CK2α level was reduced in LN18: ATM KO cells. However, XIAP depletion in LN18: ATM KO cells restored CK2α levels comparable to control cells (Figure 4I, Supplementary Figure S6I). Similarly, XIAP depletion also restored CK2α levels in ATM depleted HeLa (Figure 4J, Supplementary Figure S6J) and OVCAR3 cells (Figure 4K, Supplementary Figure S6K). In addition, as shown in Figure 4L and Supplementary Figure S6L, in LN229:ATM KO cells, p85α KD reduced CK2α and pCK2α levels but was restored to basal levels upon XIAP depletion.

Bio-informatics analysis of phosphorylation sites on CK2α and XIAP: In the current study we observed that ATM depletion reduced phosphorylation of CK2α at Thr^360^ and Ser^362^ (Figure 3A, Supplementary Figure S3A). However, bioinformatics analyses revealed that these sites (and others) are not the putative SQ/TQ ATM phosphorylation motifs, sμggesting that additional kinases regulated by ATM may be involved in the phosphorylation of CK2α. On the other hand, human XIAP had two SQ/TQ cluster domains. Interestingly, however, ATM depletion surprisingly resulted in increased phosphorylation of XIAP at Ser^87^ (Figure 4D, Supplementary Figure S6D) implying additional factors regulated by ATM (such as PP2A) may promote phosphorylation and activation of XIAP.

A model for ATM regulated PTEN degradation: We propose a model implicating a role for ATM in the regulation of PTEN level via proteasome mediated degradation (Figure 4M). Functional ATM suppresses the XIAP level by promoting its polyubiquitination and degradation. In addition, ATM may also inactivate XIAP by reducing its phosphorylation at Ser^87^. When ATM is depleted, XIAP level is increased and activated which then promotes polyubiquitination of PTEN and CK2α. p85α not only stabilises PTEN but also physically interacts with CK2α and promotes its stabilization. When p85α is lost, XIAP mediated polyubiquitination of PTEN and CK2α leads to proteasome mediated degradation.

The data presented so far provides evidence for an ATM-XIAP-PTEN-CK2α-p85α signalling network in cancer. We proceeded to evaluate if the network also influences chemotherapy response and impact on clinical outcomes in cancer patients.

**Figure 5:**
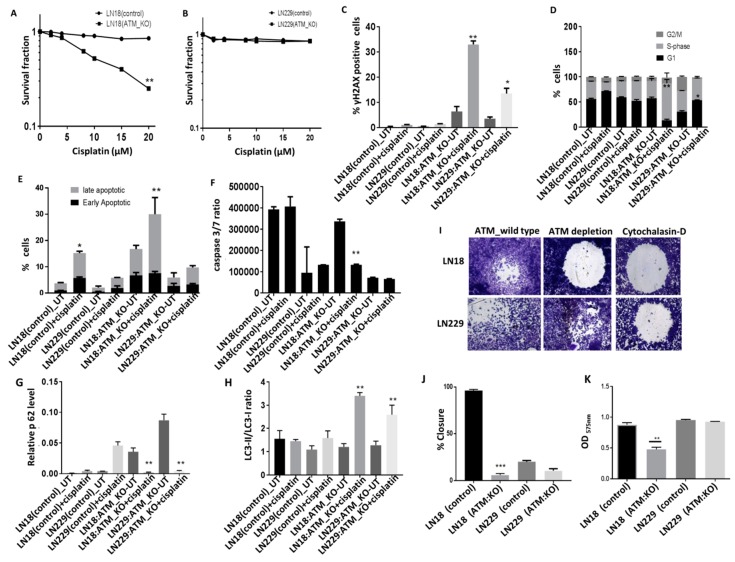
ATM depletion and platinum sensitivity. (**A**) Cisplatin sensitivity in LN18 control and ATM KO cells. (**B**) Cisplatin sensitivity in LN229 control and ATM KO cells. (**C**) LN18 control, LN229 control, LN18 ATM KO and LN229 ATM KO were treated with cisplatin for 48 h and γH2AX levels were analysed by flow cytometry. (**D**) Cell cycle analysis by flow cytometry for LN18, LN229 control and ATM KO cells treated with cisplatin. (**E**) Annexin V analysis by flow cytometry in LN18, LN229 control and ATM KO cells treated with cisplatin. (**F**) caspase 3/7 ratio by chemiluminescence detection in LN18, LN229 control and ATM KO cells treated with cisplatin. (**G**) p62 protein levels by western blotting in LN18, LN229 control and ATM KO cells treated with cisplatin. (**H**) LC3I and II protein levels by western blotting in LN18, LN229 control and ATM KO cells treated with cisplatin. (**I**) Representative photomicrographic images for migration assay for LN18and LN229 control and ATM KO cells. CytochalasinD (migration inhibitor 1.5 µM) was used as a negative control. (**J**) percentage closure quantification by imageJ software. (**K**) Invasion assay in LN18, LN229 control, and ATM KO cells. * *p* value < 0.05, ** *p* value < 0.01, *** *p* value < 0.001.

Apoptosis and autophagy in cisplatin treated ATM deficient cells: PTEN and ATM are involved in several cellular processes including cell growth, proliferation, cell-cycle progression, apoptosis, autophagy and chemotherapy response [11,17,18,19]. We tested platinum sensitivity in ATM deficient or proficient cells. As shown in Figure 5A, in p85α deficient LN18 cells, ATM KO significantly increased platinum cytotoxicity but not in p85α proficient LN229 cells (Figure 5B). Increased sensitivity to cisplatin was associated with double strand break accumulation (Figure 5C) and S-phase cell cycle arrest (Figure 5D). Given the role of XIAP in the regulation of apoptosis and autophagy caspases 3 and 7 binding [36] and a role for ATM in the regulation of cell fate [37,38], we investigated autophagy and apoptosis pathways upon cisplatin treatment. We evaluated the induction of apoptosis using annexinV-FACS (Figure 5E) in addition to Caspase 3/7 activity assay (Figure 5F). Using both approaches, we did not see any induction of early apoptotic cells (Figure 5E).IN addition, low caspase 3/7 ratio was evident following cisplatin treatment (Figure5F) in ATM KO LN18 as well as LN229 cells. For the evaluation of autophagy, p62 protein level and LC3-II/LC-I ratio were investigated. Cisplatin treatment promoted autophagy in both LN18 and LN229 ATM KO cells (Figure 5G,H), althoμgh we only observe significant induction of late apoptotic or necrotic cells in LN18 ATM KO treated with cisplatin. The data provides evidence that ATM KO promotes induction of autophagy in LN18 and LN229 cells and pro-cell death pathway upon cisplatin treatment. Interestingly, when p85α was re expressed in LN18 ATM KO cells, we observed relative resistance to cisplatin treatment (Supplementary Figure S9A). ATM activation and PI3K activation can also promote migration and invasion. We observed reduced migration (Figure 5I,J) and invasion (Figure 5K) in LN18ATM KO cells in comparison to their control cells but not in ATM KO LN229 cells.

**Figure 6:**
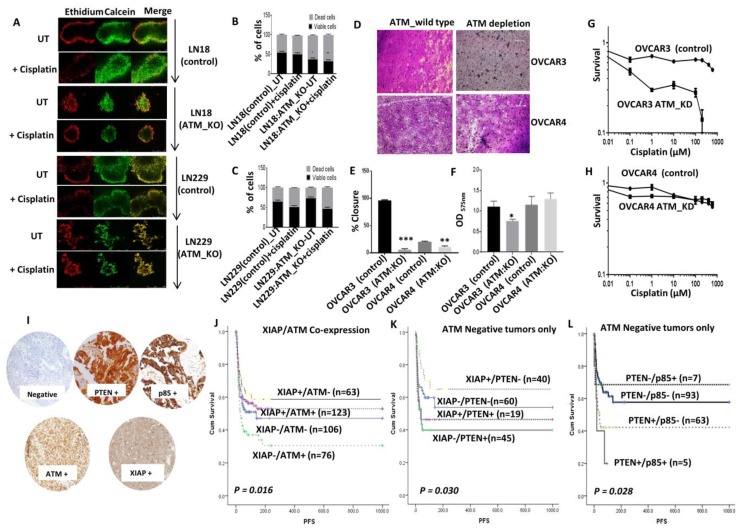
Clinicopathological significance of ATM, PTEN, p85α, and XIAP in ovarian cancers. (**A**) Representative photo micrographic images of LN18, LN229 cells control and ATM KO cells 3D-neurospheres treated with Cisplatin (40 µM). (**B**) Quantification of viable/dead cells by flow cytometry in LN18 control and ATM KO cells. (**C**) Quantification of viable/dead cells by flow cytometry in LN229 control and ATM KO. (**D**) Representative figure for Invasion assay in OVCAR3 and OVCAR4 control and ATM_KD. (**E**) Percentage closure quantification by imageJ software. (**F**) Invasion quantification in OVCAR3 and OVCAR4 control and ATM_KD. (**G**) Cisplatin sensitivity in OVCAR3 ATM_KD cells. (**H**) Cisplatin sensitivity in OVCAR4 ATM_KD cells. (**I**) Immunohistochemical expression in ovarian carcinoma TMA cores: Negative control, high PTEN, high P85, high ATM and high XIAP (200× magnification). Kaplan-Meier curve for PFS ovarian cancers of (**J**) XIAP and ATM co-expression. (**K**) XIAP and PTEN co-expression in ATM negative tumors. (**L**) PTEN and p85 co-expression in ATM negative tumours. * *p* value < 0.05, ** *p* value < 0.01, *** *p* value < 0.001.

We then generated 3D-neurospheres. A striking observation was that the spheroid forming ability of LN18 ATM KO cells and LN229 ATM KO cells were substantially impaired compared to LN18 control cells and LN229 control cells respectively (Figure 6A). In addition, cisplatin sensitivity was also evident in LN18:ATM KO neurospheres compared to controls (Figure 6B) and LN229 cells (Figure 6C). For further validation we tested ovarian cancer cell lines and investigated clinical relevance in ovarian cancer patients who received platinum based chemotherapy.

Clinical significance of ATM, PTEN, p85α, and XIAP expression in human ovarian cancers: Similar to the data shown for LN18 cells, ATM depletion in p85α deficient OVCAR3 cells was associated with reduced migration (Figure 6D,E) and invasion (Figure 6F) compared to p85α proficient OVCAR4 cells. We then tested cisplatin sensitivity in p85α deficient OVCAR3 (in control and ATM KD) and p85α proficient OVCAR4 (in control and ATM KD) cells. In OVCAR3 cells, ATM depletion resulted in substantial sensitization to cisplatin chemotherapy as assessed by clonogenic assays (Figure 6G). On the other hand, ATM depletion in OVCAR4 cells did not alter cisplatin sensitivity (Figure 6H).

We then conducted immunohistochemical investigations of ATM, XIAP, PTEN, and p85α in a large clinical cohort of 525 human epithelial ovarian cancers treated at Nottingham University Hospitals (NUH) between 1997 and 2010 (Figure 6I). All patients received platinum based chemotherapy. Progression-free survival (PFS) was calculated from the date of the initial surgery to disease progression or from the date of the initial surgery to the last date known to be progression-free for those censored. Tumours with high XIAP/low ATM levels have better PFS (*p* = 0.016) (Figure 6J) after platinum based chemotherapy. In tumours with low ATM levels, XIAP high/PTEN low tumours have improved PFS (*p* = 0.030) (Figure 6K). Similarly, in tumours with low ATM levels, PTEN low/p85α low tumours have better PFS compared to tumours with high PTEN/high p85α (*p* = 0.028) (Figure 6L). Taken together, the data provide evidence ATM-XIAP-PTEN-p85α signalling network also has clinical relevance in ovarian cancers.

## 5. Discussion

Platinum based chemotherapy has significantly improved clinical outcomes for patients with advanced ovarian cancers. Althoμgh initially chemotherapy sensitive with a response rate of about 60–80%, most ovarian tumours will develop platinum resistance and patients eventually succumb to the disease. Moreover, platinating agents related toxicities can be debilitating for patients and can include nausea, vomiting, peripheral neuropathy, nephrotoxicity, ototoxicity, and others [1,2]. Therefore, the development of biomarkers to predict sensitivity to platinum chemotherapy is highly desirable. In the current study we provide evidence that an ATM-p85α-CK2α-PTEN network operates in ovarian tumours that influence platinum sensitivity in patients. 

Whereas ATM [24], PTEN [20,21] and p85α [8,22,23] are key tumour suppressors, CK2 is frequently overexpressed in cancer and may have oncogenic function [39]. In this study, we elucidate a previously unknown network where ATM regulates PTEN degradation via XIAP E3 ubiquitin ligase in a p85α dependent manner. We also provide the first evidence that CK2α degradation is also regulated by ATM, mediated by XIAP and is also p85α dependent. The data presented here provide new insights into a complex network that may operate to not only influence cancer development and progression but also impact upon response to therapy.

Monoubiquitination and SUMOylation of PTEN control nuclear localization [7,8,9]. Recently, ATM, in response to DNA damage was also shown to phosphorylate PTEN at Ser^113^ and promote nuclear localization of PTEN [28]. As expected, in the current study, we observed loss of nuclear localization and cytoplasmic retention in ATM depleted or KU55933 treated cells. PTEN stability is regulated throμgh phosphorylation of a cluster of residues at C-terminal tail including Ser^380^, Thr^382^ and Thr^383^ [7,8,9]. Here we provide the first evidence that phosphorylation of PTEN at Ser^380^, Thr^382^ and Thr^383^ is strikingly reduced in ATM depleted or KU55933 treated cells which leads onto proteasome mediated PTEN degradation in ATM depleted or KU55933 treated cells. The data therefore sμggest that ATM promotes PTEN stability via phosphorylation of Ser^380^, Thr^382^ and Thr^383^ residues of PTEN. Whether this phosphorylation is directly or indirectly mediated by ATM is not known. As ATM phosphorylation motifs are not evident at Ser^380^, Thr^382^ and Thr^383^ residues of PTEN we speculate an indirect mechanism may be operating and would require further studies.

Previous studies sμggest that CK2 [40] and GSK3β [34] serine-threonine kinases can phosphorylate PTEN at C-terminal tail and promote PTEN stability [7]. Whereas GSK3β can phosphorylate Ser^362^, Thr^366^, CK2 is known to phosphorylate Ser^370^, Ser^380^, Thr^382^, Thr^383^ and Ser^385^ residues of PTEN [7]. Herein we show that ATM is involved in phosphorylation of CK2α sub-unit of CK2 at Thr^360^ and Ser^362^. As there are no ATM phosphorylation motifs in CK2α, we predict that additional kinases regulated by ATM may be involved in the phosphorylation of CK2α. More importantly, we also show that ATM also regulates CK2α levels throμgh proteasome mediated degradation via XIAP E3 ubiquitin ligase. Recently, IP7 (5-diphosphoinositol pentakiphosphate) was shown to bind to CK2 which in turn phosphorylates TTT (Tei 1, Tei2 and Tei3) complex. Phosphorylated TTT then binds to and stabilizes ATM [41]. In the current study, we provide evidence of a feedback loop wherein ATM itself can then regulate CK2α via XIAP mediated degradation.

Whereas mono-ubiquitination of PTEN regulates nuclear localization and has a tumour suppressing function, poly-ubiquitination targets PTEN for proteasome dependent degradation which is oncogenic [7,8,9]. E3 ubiquitin ligases are a large group of enzymes that poly-ubiquitinate target proteins [42]. ATM was previously shown to modulate the activity of several E3 ubiquitin ligases involved in DNA damage response including MDM2/MDM4 [43], Siah-1 [44], RNF20-RNF40 complex [45] and ITCH [46]. Poly-ubiquitination of PTEN can be accomplished by NEDD4-1, XIAP, WWP2, RFP, chaperone associated E3 ligase and C-terminus of CHIP [8]. XIAP is of particular interest as there may be a functional association with Akt [47] and ATM can also phosphorylate Akt [48]. The data presented here provide the first evidence that ATM regulates XIAP levels. Firstly, in ATM proficient cells, the basal level of polyubiquitinated form of XIAP was surprisingly high. Whereas in ATM KO cells, polyubiquitinated form of XIAP was low. Whilst regulation of XIAP by ubiquitnation was previously described [49], the mechanism regulating polyubiquitination is largely unknown. Our results confirm that ATM is involved in the regulation of XIAP polyubiquitnation. Whether the polyubiquitination is mediated by another E3 ubiquitin ligase or throμgh auto-ubiquitination of XIAP is currently unknown. Secondly, ATM KO surprisingly increases the level of XIAP phosphorylated at Ser 87. This paradoxical observation sμggests that additional factors regulated by ATM are involved in the phosphorylation and activation of XIAP. As PP2A phosphatase was reduced in ATM depleted cells, it is the likely mechanism for accumulation of phosphorylated form of XIAP. Althoμgh bioinformatics analysis revealed that XIAP could be subjected to extensive phosphorylation including by ATM, whether this contributes to regulation of XIAP activity remains to be established. Thirdly, XIAP depletion in ATM KO or depleted cells prevented PTEN degradation. Fourthly, ATM regulation of CK2α degradation is also XIAP mediated. Finally, XIAP accumulation in ATM KO cells results in polyubiquitination of PTEN leading to proteasome mediated degradation.

An important observation in the current study is that ATM regulated PTEN degradation is p85α dependent. In p85α proficient cells, ATM depletion or KU55933 treatment did not lead to PTEN degradation. When p85α was depleted, ATM KO/KD or KU55933 treatment leads to degradation of PTEN. Previously, p85α was shown to bind to PTEN and prevent polyubiquitination of PTEN [22,23,29]. A new observation in the current study is that p85α also binds to CK2α and prevents degradation mediated via XIAP in ATM deficient cells.

ATM inhibition has recently emerged as a promising anti-cancer strategy [50]. In addition, ATM targeting also has the potential to enhance the cytotoxicity of chemotherapy. We demonstrate cisplatin chemo-sensitivity in p85α deficient/ATM deficient cells but not in in p85α proficient/ATM deficient cells, a phenotype that is reversed upon ectopic p85α expression in previously deficient cells. This increased sensitivity is predominantly mediated via the induction of autophagy. Althoμgh autophagy has been described as cell survival mechanism, emerging evidence also sμggest a pro-cell death function for autophagy. Given a previously described role for ATM in radiation induced autophagy [37], we speculate a novel role for ATM in autophagy regulation via interaction with XIAP. However further functional studies are required to confirm this hypothesis. Interestingly, 3D-neurosphere forming ability is severely impaired in ATM:KO cells, a feature that would concur with a previous study demonstrating a role for ATM in the re-constitutive capacity of haematopoetic stem cells [51]. In addition, cisplatin sensitivity was confirmed in 3D-models. In clinical cohorts of human ovarian cancers, we have demonstrated ATM, PTEN, p85α and XIAP expression as predictors of response to platinum chemotherapy. 

In conclusion, the data presented here reveal a novel ATM-p85α-XIAP-PTEN network with translational applications in cancer.

## Supplementary Materials

The following are available online at https://www.mdpi.com/2073-4409/8/10/1271/s1. **Table S1:** Patient demographics and pathological features in ovarian cancer; **Table S2:** Antigens, primary antibodies, clone, source, optimal dilution and scoring system used for each immunohistochemical marker; **Figure S1:** Relative protein quantification by western blotting of (**A**) ATM, PTEN and p-AKT1in LN18 and LN229cells. (**B**) ATM, PTEN and p-PTEN in LN18 control and ATM_KO. (**D**) PTEN and p-PTEN in LN18 untreated and KU55933 treated cells; **Figure S2:** Relative protein quantification by western blotting of (**A**) PTEN and p-PTEN in LN18 control and ATM_KO cells treated LN18 with cycloheximide and MG132 as indicated. (**D**) p-AKT1levels in LN18 control and ATM_KO cells. (**E**) PTEN levels in LN18 cells treated with pan AKT inhibitor MK2206. (**G**) PTEN and p-PTEN in LN18p85_KI control and LN18 p85_KI KU55933 treated cells. (**H**) ATM, PTEN and p-PTEN in LN229 control and ATM _KO cells. (**I**) PTEN and p-PTEN in KU55933 treated LN229 cells; **Figure S3:** (**A**) CK2α and p-CK2α in LN18 control and ATM_KO cells. (**B**) PTEN and p-PTEN in LN18 CK2_KD cells. (**C**) GSKβ and p-GSKβ in LN18 control and ATM_KO cells. (**D**) CK2α and p-CK2α in Hela control and ATM_KO cells. (**E**) PTEN and p-PTEN in CK2_KD Hela cells. (**F**) PTEN and p-PTEN in OVCAR3 CK2_KD cells. (**G**) CK2α and p-CK2α in LN229 ATMKO cells. (**H**) CK2α and p-CK2α in KU55933 treated LN229 cells. (**I**) PTEN and p-PTEN in LN18 p85_KI cells CK2α knock down (**K**) PTEN and p-PTEN in CK2_KD, p85 +CK2_KD and p85 _KD+ CK2inhibitor LN229 cells. (**L**) CK2α and p-CK2α in OVCAR4 ATM_KD cells. (**M**) CK2α and p-CK2α in KU55933 treated OVCAR4 cells. (**R**) PTEN and p-PTEN in OVCAR4 in p85_KD, p85_KD+ CK2_KD andp85_KD +CK2 inhibitor treated OVCAR4 cells; **Figure S4:** (**A**) Levels of ATM, PTEN and p-PTEN and p85α in Hela ATM SilenciX cells. As well as levels of PTEN and p-PTEN in Hela control cells treated with 10 μM KU55933. (**B**) PTEN levels in OVCAR3 and OVCAR4 cell lines.PTEN and p-PTEN levels in ATM depleted OVCAR3 cells. PTEN and p-PTEN levels in OVCAR3 were treated with 10 μM of KU55933. (**C**) PTEN and p-PTEN levels in OVCAR4 cells transfected with ATM SiRNA.Levels of PTEN and p-PTEN in OVCAR4 treated with 10 μM of KU55933; **Figure S5:** (**A**) CK2α, p-CK2α levels in LN18 untreated and KU55933 treated cells. (**B**) PTEN, p-PTEN levels in LN18 cells treated with CK2α inhibitor. (**C**) CK2α and p-CK2α levels in Hela control cells treated with KU55933. (**D**) Levels of PTEN, p-PTEN in Hela cells treated with CK2α inhibitor. (**E**) PTEN, p-PTEN and CK2α levels in OVCAR3 cells treated with CK2α inhibitor; **Figure S6:** (**D**) p-XIAP levels in LN18 and LN229 control and ATM_KO cells. (**E**) PTEN and p-PTEN in ATM_KO, ATM_KO+XIAP KD LN18 cells. (**F**) PTEN and p-PTEN in ATM_KD alone and ATM_KD +XIAP double KD in Hela cells. (**G**) PTEN and p-PTEN in ATM_KD alone and ATM_KD +XIAP double KD OVCAR3 cells. (**H**) CK2α in ATM _KO and ATM_KO +XIAP_KD in LN18 cells. (**I**) CK2α in ATM_KD and ATM_KD +XIAP_KD in Hela cells. (**J**) CK2α in ATM_KD and ATM_KD+XIAP _KD OVCAR3 cells. (**K**) CK2α and p-CK2α in LN229 ATM_KO+ p85_KD and ATM_KO+p85 and XIAP double KD LN229 cells; **Figure S7: (A)** PTEN and p-PTEN in LN18 control cells treated with KU55933 and treated accordingly with cycloheximide and MG132 for the different time points. (**B**) PTEN and p-PTEN in LN18 p85 KI cells treated with KU55933. (**C**) Doxycycline inducible PTEN in LN229 cells; **Figure S8: (A)** ATM, PTEN and p-PTEN in Hela control and ATM_KD cells. PTEN and p-PTEN in Hela control untreated and KU55933 treated cells. (**B**) PTEN in OVCAR3 and OVCAR4. (**C**) PTEN and p-PTEN in OVCAR3 control and OVCAR3 ATM_KD. PTEN and p-PTEN in OVCAR3 untreated and KU55933 treated cells. (**D**) ATM, PTEN and p-PTEN in OVCAR4 ATM_KD cells. PTEN and p-PTEN in KU55933 treated OVCAR4 cells. (**E**) CK2α and p-CK2α in KU55933 treated LN18. (**F**) PTEN and p-PTEN in CK2 inhibitor treated LN18 cells. (**G**) CK2α and p-CK2α in Hela control cells treated with KU55933. PTEN and p-PTEN in CK2 inhibitor treated Hela cells. (**H**) PTEN and p-PTEN in CK2 inhibitor treated OVCAR3 cells; **Figure S9:** (**A**) Cisplatin sensitivity in LN18 ATM_KO and LN18 ATM_KO_p85 KI. (**B**) PTEN and p-PTEN in p85_KD, ATM_KD, p85+ATM _KD and p85 +KU55933 in OVCAR4 cells. (**C**) PTEN and p-PTEN in OVCAR4 in p85_KD, p85_KD+ CK2_KD andp85_KD +CK2 inhibitor treated OVCAR4 cells. (**D**) Kaplan-Meier curve for PFS ovarian cancers of: XIAP/ PTEN co-expression in the whole cohort. XIAP /PTEN co-expression in ATM positive tumours only. PTEN/p85 co-expression in ATM positive tumours only; **Figure S10: (A)** Potential phosphorylation sites for ATM on XIAP. (**B**) Amino acids translation of human CK2α sequence.

## Abbreviations

ATM: X-ray repair cross-complementing gene 1; PTEN: Phosphatase and tensin homolog; PIP_3_: phosphatidylinositol (3,4,5)-triphosphate; PIP_2_: phosphatidylinositol 4,5) triphosphate; ATM: Ataxia-telegiectasia mutated; DSB: double strand breaks (DSBs); CK2: Casein Kinase 2; KO: knock out; TMA: tissue microarray; IHC: immunohistochemistry.
