# Supplementary material for: ATM Regulated PTEN Degradation Is XIAP E3 Ubiquitin Ligase Mediated in p85α Deficient Cancer Cells and Influence Platinum Sensitivity
Source: Cells. 2019 Oct 18;8(10):1271. doi: 10.3390/cells8101271 (PMC6848936; doi:10.3390/cells8101271)
Supplement: Supplementary file 1 [file cells-08-01271-s001.pdf]

Supplementary Figure legends

Supplementary figure S1: Relative protein quantification by western blotting of **(A)** ATM, PTEN and p-AKT1in LN18 and LN229cells. **(B)** ATM, PTEN and p-PTEN in LN18 control and ATM_KO. **(D)** PTEN and p-PTEN in LN18 untreated and KU55933 treated cells.

Supplementary Figure S2: Relative protein quantification by western blotting of **(A)** PTEN and p-PTEN in LN18 control and ATM_KO cells treated LN18 with cycloheximide and MG132 as indicated. **(D)** p-AKT1levels in LN18 control and ATM_KO cells. **(E)** PTEN levels in LN18 cells treated with pan AKT inhibitor MK2206. **(G)** PTEN and p-PTEN in LN18p85_KI control and LN18 p85_KI KU55933 treated cells. **(H)** ATM, PTEN and p-PTEN in LN229 control and ATM _KO cells. **(I)** PTEN and p-PTEN in KU55933 treated LN229 cells.

Supplementary Figure S3: **(A)** CK2α and p-CK2α in LN18 control and ATM_KO cells. **(B)**.PTEN and p-PTEN in LN18 CK2_KD cells. **(C)** GSKβ and p-GSKβ in LN18 control and ATM_KO cells. **(D)** CK2α and p-CK2α in Hela control and ATM_KO cells.. **(E)** PTEN and p-PTEN in CK2_KD Hela cells. **(F)** PTEN and p-PTEN in OVCAR3 CK2_KD cells. **(G)** CK2α and p-CK2α in LN229 ATMKO cells. **(H)** CK2α and p-CK2α in KU55933 treated LN229 cells. **(I)** PTEN and p-PTEN in LN18 p85_KI cells CK2α knock down **(K)** PTEN and p-PTEN in CK2_KD, p85 +CK2_KD and p85 _KD+ CK2inhibitor LN229 cells. **(L)** CK2α and p-CK2α in OVCAR4 ATM_KD cells. **(M)** CK2α and p-CK2α in KU55933 treated OVCAR4 cells. **(R)** PTEN and p-PTEN in OVCAR4 in p85_KD, p85_KD+ CK2_KD andp85_KD +CK2 inhibitor treated OVCAR4 cells.

Supplementary Figure S4: **(A)** Levels of ATM, PTEN and p-PTEN and p85α in Hela ATM SilenciX cells. As well as levels of PTEN and p-PTEN in Hela control cells treated with 10µM KU55933. **(B)** PTEN levels in OVCAR3 and OVCAR4 cell lines.PTEN and p-PTEN levels in ATM depleted OVCAR3 cells. PTEN and p-PTEN levels in OVCAR3 were treated with 10µM of KU55933. **(C)** PTEN and p-PTEN levels in OVCAR4 cells transfected

with ATM SiRNA.Levels of PTEN and p-PTEN in OVCAR4 treated with 10µM of KU55933.

Supplementary Figure S5: (A) CK2α, p-CK2α levels in LN18 untreated and KU55933 treated cells. **(B)** PTEN, p-PTEN levels in LN18 cells treated with CK2α inhibitor. **(C)** CK2α and p-CK2α levels in Hela control cells treated with KU55933. **(D)** Levels of PTEN, p-PTEN in Hela cells treated with CK2α inhibitor. **(E)** PTEN, p-PTEN and CK2α levels in OVCAR3 cells treated with CK2α inhibitor**.**

Supplementary Figure S6: (D) p-XIAP levels in LN18 and LN229 control and ATM_KO cells. **(E)** PTEN and p-PTEN in ATM_KO, ATM_KO+XIAP KD LN18 cells.**(F)** PTEN and p-PTEN in ATM_KD alone and ATM_KD +XIAP double KD in Hela cells. **(G)** PTEN and p-PTEN in ATM_KD alone and ATM_KD +XIAP double KD OVCAR3 cells. **(H)**CK2α in ATM _KO and ATM_KO +XIAP_ KD in LN18 cells. **(I)** CK2α in ATM_KD and ATM_KD +XIAP_KD in Hela cells. **(J)** CK2α in ATM_KD and ATM_KD+XIAP _KD OVCAR3 cells. **(K)** CK2α and p-CK2α in LN229 ATM_KO+ p85_KD and ATM_KO+p85 and XIAP double KD LN229 cells.

Supplementary Figure S7: (A) PTEN and p-PTEN in LN18 control cells treated with KU55933 and treated accordingly with cycloheximide and MG132 for the different time points. **(B)** PTEN and p-PTEN in LN18 p85 KI cells treated with KU55933. **(C)**Doxycycline inducible PTEN in LN229 cells.

Supplementary Figure S8: (A) ATM, PTEN and p-PTEN in Hela control and ATM_KD cells. PTEN and p-PTEN in Hela control untreated and KU55933 treated cells.**(B)**PTEN in OVCAR3 and OVCAR4 .**(C)** PTEN and p-PTEN in OVCAR3 control and OVCAR3 ATM_KD. PTEN and p-PTEN in OVCAR3 untreated and KU55933 treated cells. **(D)** ATM, PTEN and p-PTEN in OVCAR4 ATM_KD cells. PTEN and p-PTEN in KU55933 treated OVCAR4 cells. **(E)** CK2α and p-CK2α in KU55933 treated LN18. **(F)** PTEN and p-PTEN in CK2 inhibitor treated LN18 cells. **(G)** CK2α and p-CK2α in

Hela control cells treated with KU55933. PTEN and p-PTEN in CK2 inhibitor treated Hela cells. **(H)** PTEN and p-PTEN in CK2 inhibitor treated OVCAR3 cells.

Supplementary Figure S9: (A) Cisplatin sensitivity in LN18 ATM_KO and LN18 ATM_KO_ p85 KI. **(B)** PTEN and p-PTEN in p85_KD, ATM_KD, p85+ATM _KD and p85 +KU55933 in OVCAR4 cells. **(C)** PTEN and p-PTEN in OVCAR4 in p85_KD, p85_KD+ CK2_KD andp85_KD +CK2 inhibitor treated OVCAR4 cells. **(D)** Kaplan-Meier curve for PFS ovarian cancers of: XIAP/ PTEN co-expression in the whole cohort. XIAP /PTEN co-expression in ATM positive tumours only. PTEN/p85 co-expression in ATM positive tumours only.

Supplementary Figure S10: (A) Potential phosphorylation sites for ATM on XIAP. **(B)** Amino acids translation of human CK2α sequence.
